# Shade and nutrient-mediated phenotypic plasticity in the miracle plant *Synsepalum dulcificum* (Schumach. & Thonn.) Daniell

**DOI:** 10.1038/s41598-019-41673-5

**Published:** 2019-03-26

**Authors:** Dèdéou A. Tchokponhoué, Sognigbé N’Danikou, Jacob S. Houéto, Enoch G. Achigan-Dako

**Affiliations:** Laboratory of Genetics, Horticulture and Seed Science (GBioS), Faculty of Agronomic Sciences (FSA), University of Abomey-Calavi (UAC), Abomey-Calavi, Republic of Benin

## Abstract

Phenotypic plasticity as a change of genotype expression in response to environmental heterogeneity varies in magnitude among crop species and can induce a shift in a plant’s phenology. In *Synsepalum dulcificum*, a West African orphan fruit tree, such phenological plasticity is not well understood. Here, we hypothesize that light stimulation and changes in organic nutrient availability would induce an accelerated transition in *S. dulcificum* from its juvenile to its reproductive phase. We grew 14-month-old seedlings of *S. dulcificum* under a range of nutrient regimes, both in shade and in full sunlight, and measured their survival, vegetative growth, biomass allocation, and transition to reproductive maturity. The results reveal that *S. dulcificum* responds favourably to both shading and nutrient application, with the shading exhibiting a stronger influence on the measured variables. The species’ morphological plasticity, particularly in terms of plant height and stem diameter, was found to exceed both its fitness and allocational plasticities. Under the conditions examined, we observed an accelerated transition to fruiting, at an age of only 24 months. The observed plasticity suggests *S. dulcificum* to be an intermediate shade-tolerant species. This finding expands our knowledge on the appropriate environmental conditions for the breeding and cultivation of this species.

## Introduction

Phenotypic plasticity refers to the alteration of an individual’s morphology, physiology, development, and/or life history in response to environmental heterogeneity^1,2^. For plant species, it is the means by which an individual adjusts to environmental changes and optimizes resource acquisition. Likely to have ecological, horticultural, evolutionary, and fitness implications^3–5^, phenotypic plasticity plays a key role in the ecological expansion of invasive species^6,7^. In horticultural crops, it can be exploited to improve productivity^8^ and is an important consideration for breeders wrestling with genetic correlation among important morphological traits^9^.

Historically, light, water, nutrient, temperature, and wind were the common environmental factors for which phenotypic plasticity was evaluated in plant species^4,10–13^; and the degree of plasticity was consistently found to vary, depending on both the species and the traits under consideration^4,13–17^. For instance, *Pistacia lentiscus* L., and *P. terebinthus* L. were more plastic than *Quercus coccifera* L. and *Q. faginea* Lam. in their response to irradiance, whereas *Quercu*s spp. were more plastic than *Pistacia* spp. in their response to water availability^18^. Likewise, it has been suggested that light-demanding species exhibit greater plasticity in growth, morphology, and physiological traits than shade-tolerant species^19^; and in liana species (e.g. *Rosa longicuspis* Bertol*., Embelia procumbens* Hemsl.), biomass and growth traits were found to be more plastic in response to altered light than morphological traits^20^. In *Populus deltoides* Bartr. ex Marsh., altered nutrient availability was associated with greater plasticity in shoot-specific traits than in leaf-specific traits, whereas for the same species the relative magnitude of the plasticity in the two sets of traits was similar in response to changes in water availability^21^. Likewise, a recent study comparing seven domesticated crops to their respective wild relatives highlighted a differential plasticity pattern to nutrient and water availability for a suite of traits, including maximum height, total leaf area, plant-level photosynthetic rate, and growth performance traits^14^. In another study, altered nutrient availability induced plasticity in both leaf number and total leaf area in *Pelargonium australe* J. Jacq.^22^. Such findings suggest that factors like light, water, and nutrient availability do not trigger a predictable plasticity pattern across all plant species. In general, understanding such species-specific plasticity patterns is necessary to inform production techniques and optimize trait selection. Thus, for the miracle plant *Synsepalum dulcificum* (Schumach & Thonn.) Daniell, an endangered orphan fruit species in which fast growth, early fruiting, and higher yields are desired, basic investigation of these issues is needed.

*Synsepalum dulcificum* is an evergreen West African native species belonging to the Sapotaceae family. This tropical species is the only known natural source of “miraculin”, a sweetening glycoprotein^23^. The fruit (miracle berry) has been reported as a promising economic alternative to synthetic sugar^24,25^ and is recommended for diabetic patients^26^. Currently, *S. dulcificum* is utilised in cosmetics and food, though it is most extensively used by the pharmaceutical industry^27^. Interest in the species has grown tremendously in the last decade, with one kilogram of pure powder of the fruit fetching prices of up to $2,500 (http://miraclefruitfarm.com/shop/). Developing early fruiting and highly productive ecotypes for large-scale production will be crucial for meeting this increasing demand. To date, however, *S. dulcificum* is a notoriously slow-growing species^28^, often encountered in home gardens and less frequently in farms^29^. Empirical observations have suggested that seedlings growing in home, tree-based gardens, are more vigorous than those exposed to full sunlight on open farms. It is therefore important to know whether or not irradiance reduction is beneficial to the species and, if so, to what extent.

Wilkie *et al*.^30^ indicated environmental induction as an appropriate means of inducing flowering in various tropical horticultural crops. Meilan^31^ suggested that photoperiod, nutrient, availability, and water were the main environmental factors that trigger flowering in woody species. For example, light exposure consistently accelerated flowering in the blueberry (*Vaccinium corymbosum* L.)^32^, while in the white birch (*Betula platyphylla* Suk.) a balanced NPK fertilization was reported not only to promote the transition from the juvenile stage to maturity, but also to increase flower production^33^. In addition, in the case of *S. dulcificum*, irrigation and inorganic fertilization have previously been reported to accelerate and increase flowering^34^, though whether or not the source of the fertilization mattered for growth and development remains unknown. While a number of studies dealing with organic fertilization in tree species report beneficial effects on soil conditions, effects on the trees’ intrinsic performances (e.g. survival, growth, and reproduction) are less clear^35,36^. In *S. dulcificum*, no study has yet evaluated the response of seedlings to light exposure and organic nutrient supply. Nutrient availability and light have also been reported as important factors influencing biomass allocation, thus playing a key role in the timing of growth and reproduction^37^; however, biomass allocation pattern in *S. dulcifucm* has also yet to be investigated.

The objective of this study is to evaluate the response of *S. dulcificum* to changes in light exposure and organic nutrient supply (i.e. compost application) in an effort specifically to identify the potential of these factors to induce rapid growth and early fruiting in the species. Since *S. dulcificum* is a tropical species, we hypothesize that the plasticity of its phenotype to light and compost application will be enough to realize significant gains in both vegetative growth and early flowering.

## Results

### Effect of light exposure and compost application on seedling survival

**Figure 1:**
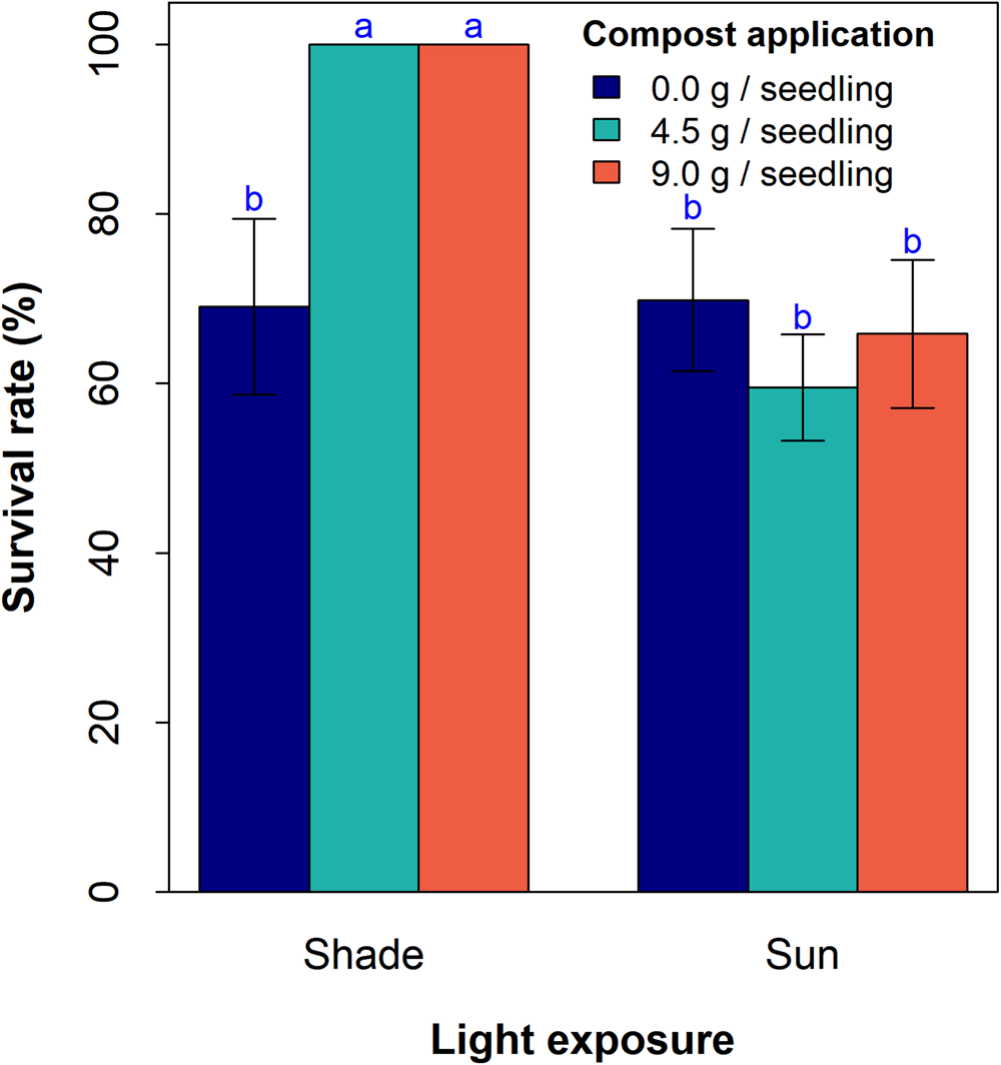
Effect of light exposure and compost application on seedling survival in *Synsepalum dulcificum*. Barplots (Treatments) with the same letter are not statistically different at 5% (Least Significant Difference post-hoc test). n = 20 seedlings and error bars represent standard error.

The results indicate that shading significantly improves seedling survival in *S. dulcificum* (Fig. 1, *p* = *0.002*). While no main effect of compost application on survival was detected (*p* > *0.05*), we did observe a significant interaction between light exposure and compost application (Fig. 1, *p* = *0.002*), such that a significant effect of compost application was detected under shade conditions. Likewise, for the same compost dosage, survival was greater in shaded seedlings than in full sun-exposed seedlings.

### Vegetative growth in response to light exposure and compost application

**Figure 2:**
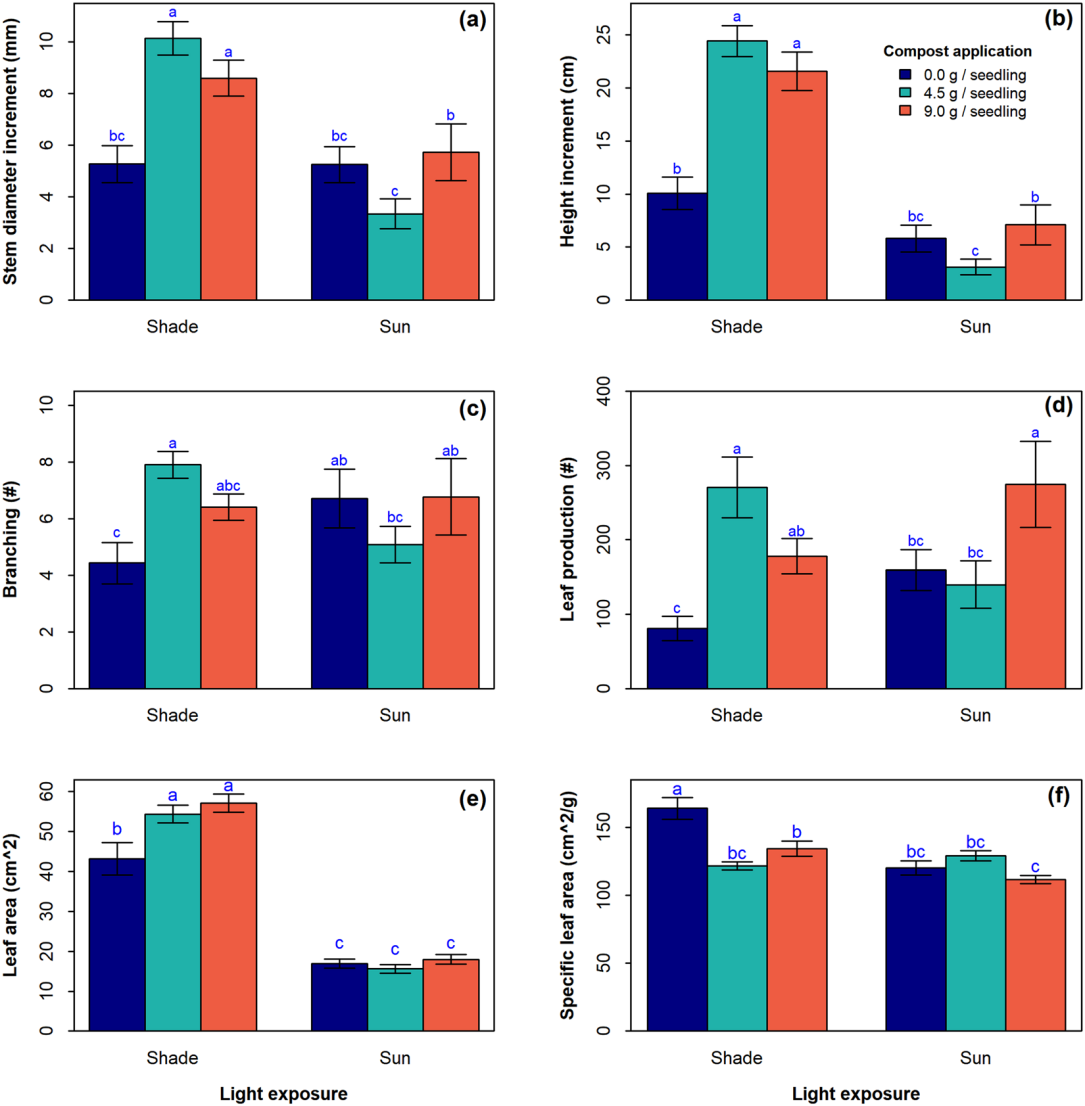
Vegetative growth of *Synsepalum dulcificum* in response to light exposure and compost application. **(a)** Diameter growth. **(b)** Height growth. **(c)** Branches production. **(d)** Leaf production. **(e)** Leaf area. **(f)** Specific leaf area. Barplots (Treatments) with the same letter are not statistically different at 5% (Least Significant Difference post-hoc test). n = 20 seedlings and error bars represent standard error.

Shading significantly increased nearly all measured aspects of seedling growth, including stem diameter (Fig. 2a, *p* < *0.001*), plant height (Fig. 2b, *p* < *0.001*), branching (Fig. 2c, *p* = *0.03*), leaf area (Fig. 2e, *p* < *0.001*), and specific leaf area (Fig. 2f, *p* < *0.001*); but there was no significant effect of light exposure on leaf production (Fig. 2d, *p* > *0.05*). Likewise, compost application positively affected stem diameter (Fig. 2a, *p* = *0.006*), plant height (Fig. 2b, *p* < *0.001*), branching (Fig. 2c, *p* = *0.002*), and specific leaf area (Fig. 2f, *p* = *0.03*); however, increased dosage did not induce a significant increased effect on those growth parameters (*p* > *0.05*). Contrary to light exposure, compost application did not affect leaf area (Fig. 2e, *p* = *0.19*), though it did affect leaf production (Fig. 2d, *p* = *0.003*). Here also, an increase in compost dosage did not translate into significantly higher leaf production (*p* = *0.87*). The interaction between light exposure and compost application was significant for all parameters except leaf area (Fig. 2). Overall, the beneficial effect of compost application on vegetative growth traits was more prominent under shade versus full sunlight.

### Reproductive growth in response to light exposure and compost application

Table 1 presents the percentages of budding, flowering, and fruit-bearing seedlings within each of the six treatments. Shading significantly enhanced budding (*p* < *0.001*), flowering (*p* = *0.006*), and fruiting (*p* = *0.02*). Likewise, compost application also significantly improved budding (*p* < *0.002*), flowering (*p* = *0.02*), and fruiting (*p* = *0.04*). However, increasing compost application beyond 4.5 g per plant had no significant effect on plant phenological transition (*p* > *0.05*). At the end of the experiment (17 months days after transplanting), only shaded and fertilized seedlings bore fruit (*p* < *0.01*).

**Table 1:** Effect of light exposure and compost application on budding, flowering, and fruit bearing in *Synsepalum dulcificum* seedlings.

Light exposure	Compost application (g/seedling)	Budding (%)	Flowering (%)	Fruiting (%)
Shade	0	0^a^ (0)	0^a^ (0)	0^a^ (0)
	4.5	45^b^ (11.41)	30^b^ (10.51)	25^b^ (9.93)
	9.0	40^b^ (11.23)	25^b^ (9.93)	25^b^ (9.93)
Sun	0	0^a^ (0)	0^a^ (0)	0^a^ (0)
	4.5	0^a^ (0)	0^a^ (0)	0^a^ (0)
	9.0	5^a^ (5)	5^a^ (5)	0^a^ (0)

Values are means (S.E.). n = 20 seedlings. Means followed by the same letter in a column are not statistically different at 5% (Least Significant Difference post-hoc test). Abbreviations: S.E (Standard error).

The times to first budding, flowering, and fruiting are presented in Table 2. While compost application was found to accelerate the transition to reproductive growth, no significant difference was observed in the times to first budding and flowering for plants that received different compost doses (*p* > *0.05*). In contrast, the time to fruiting was significantly lower in plants that received 4.5 g compost compared with those fertilized with 9.0 g compost. Overall, treated plants (shaded and fertilized) entered reproductive phase at an average age of 23 months (19 months for early-maturing individuals); and fruit-bearing was observed at an average age of 26 months (24 months for early-maturing individuals).

**Table 2:** Effect of light exposure and compost application on the timing of reproduction in *Synsepalum dulcificum* seedlings.

Light exposure	Compost application (g/seedling)	Time to first budding (days)			Time to first flowering (days)			Time to first fruiting (days)		
		Mean (SE)	Min	Max	Mean (SE)	Min	Max	Mean (SE)	Min	Max
Shade	0	—	—	—	—	—	—	—	—	—
	4.5	260.66^a^ (22.69)	160	413	282.33^a^ (16.29)	209	330	323.2^b^ (19.08)	297	399
	9.0	307.37^a^ (20.69)	246	399	329.40^a^ (22.78)	260	390	387.6^a^ (13.93)	354	422
Sun	0	—	—	—	—	—	—	—	—	—
	4.5	—	—	—	—	—	—	—	—	—
	9.0	286 (NA)	—	—	335 (NA)	—	—	—	—	—

Values are means (S.E.). n = 20 seedlings. Means followed by the same letter in a column are not statistically different at 5% (Least Significant Difference post-hoc test). Abbreviations: - (no individuals reaching the target stage); NA (not calculated as there was only one individual); Min (Minimum); Max (Maximum); S.E (Standard error).

### Biomass allocation in response to light exposure and compost application

The observed pattern of biomass partitioning within the *S. dulcificum* seedlings indicates a significant effect of light on all parameters (Tables 3 and 4). Leaf mass fraction (LMF) (*p* = *0.007*) and stem mass fraction (SMF) (*p* = *0.01*) were significantly higher in shaded plants than in those grown in full sunlight. In contrast, root mass fraction (RMF) (*p* = *0.001*) and the root-to-shoot ratio (R/S) (*p* < *0.001*) were significantly higher in seedlings grown in full sunlight. None of the tested biomass allocation parameters was significantly affected by compost application (*p* > *0.05*). Similarly, no significant interaction between light exposure and compost application was detected for any of the biomass allocation parameters (RMF, SMF, LMF, or R/S) (*p* > *0.05*) (Table 4).

**Table 3:** Biomass allocation in *Synsepalum dulcificum* seedling growing under shade and full sunlight and different compost application doses.

Light exposure	Compost application (g/seedling)	Leaf mass fraction (LMF)	Stem mass fraction (SMF)	Root mass fraction (RMF)	Root to shoot ratio (R/S)
Shade	0	0.31 (0.01)	0.29 (0.01)	0.39 (0.01)	0.66 (0.05)
	4.5	0.29 (0.01)	0.34 (0.01)	0.36 (0.02)	0.58 (0.06)
	9.0	0.339 (0.02)	0.29 (0.02)	0.34 (0.04)	0.56 (0.13)
Sun	0	0.26(0.02)	0.24 (0.007)	0.58 (0.03)	0.96 (0.12)
	4.5	0.29 (0.02)	0.24 (0.03)	0.46 (0.05)	0.91 (0.19)
	9.0	0.28(0.03)	0.24 (0.01)	0.46 (0.02)	0.87 (0.19)

Values are means (S.E.). n = 3 seedlings. Statistical analyses are shown in Table 4. Abbreviations: S.E (Standard error).

**Table 4:** ANOVA results for each biomass allocation parameter (LMF, SMF, RMF, and R/S).

Biomass allocation parameters	Light exposure (LE)	Compost application (D)	(LE) X (D)
Leaf mass fraction (LMF)	11.26^**^	0.23^ns^	01.62^ns^
Stem mass fraction (SMF)	8.3^*^	0.54^ns^	0.44^ns^
Root mass fraction (RMF)	18.95^**^	0.45^ns^	0.68^ns^
Root to shoot ratio (R/S)	25.10^**^	0.36^ns^	0.27^ns^

Values are F statistics. Significance values are represented as follow: ns = not significant; *p < 0.05; and **p < 0.01.

### Phenotypic plasticity in *S. dulcificum*

**Figure 3:**
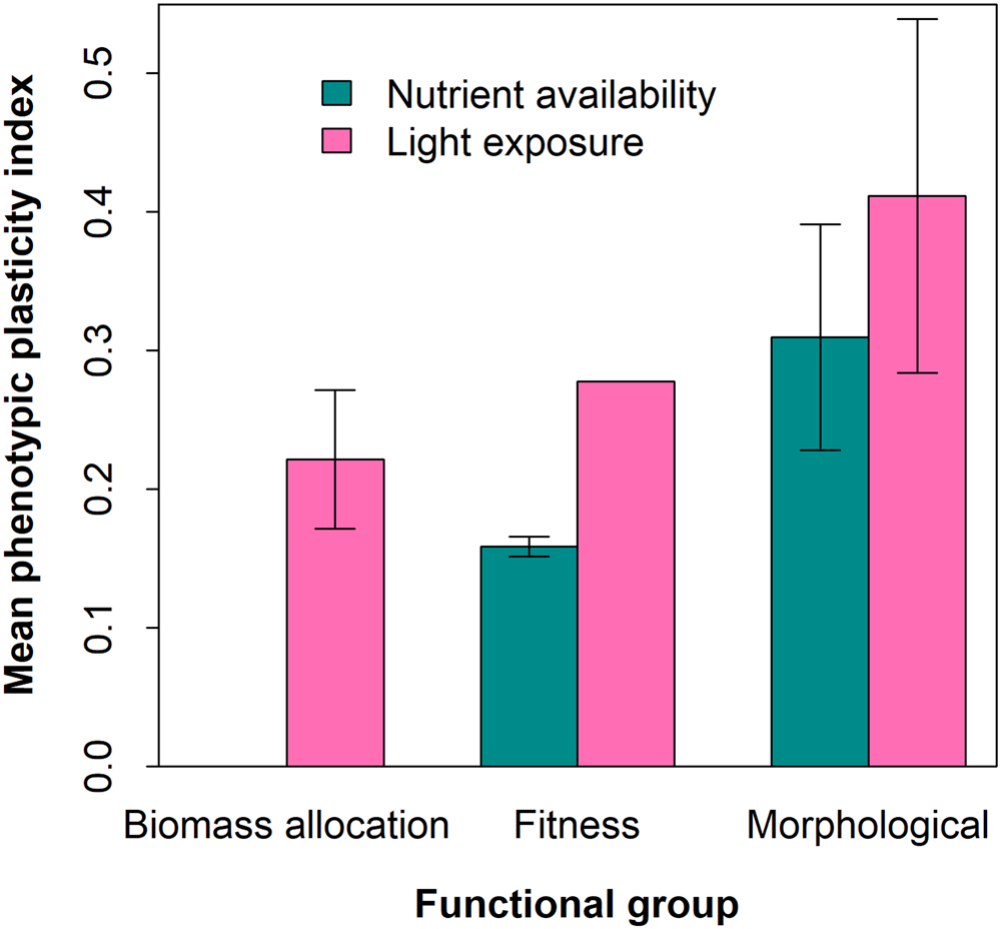
Mean phenotypic plasticity indices, by functional groups, for *Synsepalum dulcificum*.

As reported in Table 5, phenotypic plasticity indices (PPI) in *S. dulcificum* in response to light exposure and nutrient availability (compost application) ranged from 0.03 to 0.72. Of the twelve traits measured, ten were found to respond to light exposure, whereas seven were responsive to nutrient availability. The variation intensity differed greatly among quantitative traits (*p* = *0.02*). The top three varying quantitative traits to light exposure included plant height, leaf area, and stem diameter, whereas the three most varying quantitative traits to nutrient availability were plant height, number of leaves, and stem diameter. Overall, *S. dulcificum* showed a greater plasticity to changes in light intensity than to nutrient availability. From a functional point of view, *S. dulcificum* exhibited allocational plasticity only to light exposure; and in both morphological and fitness functional groups, the mean PPI was higher to light exposure than to nutrient availability (Fig. 3). The light-induced phenotypic plasticity also induced variation in leaf color, with shaded seedlings being greener than those grown under full sunlight. An illustration of the response of leaf colour to varying light exposure is shown in Supplementary Fig. S1.

**Table 5:** Phenotypic plasticity index (based on Valladares *et al*.^81^) in S*ynsepalum dulcificum* measured traits for light exposure and nutrient availability factors.

Traits	Light exposure	Nutrient availability
Height	0.72	0.51
Diameter	0.41	0.30
Branching	0.03	0.18
Leaf production	—	0.45
Leaf area	0.64	—
Specific leaf area	0.23	0.08
Stem mass fraction	0.22	—
Leaf mass fraction	0.09	—
Root mass fraction	0.21	—
Root to shoot ratio	0.34	—
Fruiting time	—	0.16
Survival	0.27	0.15
**Mean**	**0.32**	**0.26**

Abbreviations: - (no plasticity of the trait for the treatment under consideration).

## Discussion

Our study highlighted the beneficial effect of light exposure and compost application in *S. dulcificum*, a tropical tree of the Sapotaceae family. Such investigations are scanty in that family^7^, particularly in the *Synsepalum* genus, although previous studies have documented the growth of perennial species in response to shading and fertilization^23,38^.

*Synsepalum dulcificum* exhibited a relatively higher survival rate under shade conditions, as well as with ample water and nutrient supply. The beneficial effect of continuous water supply on the tree was previously known^34^. Our findings indicate that moderate shade is favourable for the survival of transplanted seedlings. The survival and growth rates obtained under shade were indicative of the sensitivity of *S. dulcificum* to heat. This observation partly explains the scarcity of the species, especially seedlings, in open field (e.g. farms, fallows). According to Grubb^39^, a shortage of light in plant species may compromise the survival of plants. However, in *S. dulcificum* the survival of seedlings was improved with a substantial reduction of sunlight. Higher juvenile survival rates under shade conditions were reported to characterize either intermediate- shade tolerant or shade tolerant species^40^. A similar phenomenon was also reported in *Ilex aquifolium* L.^41^ (another shrub species from Northern Africa, Western Asia, and Europe reported to exhibit shade tolerance).

Our findings indicate that shade also improved reproductive performance by reducing the time to first fruiting and increasing the proportion of seedlings entering reproductive phase. This finding is new in *S. dulcificum* and complements existing knowledge on tree species. Previous studies that assessed the effect of shading on tree species focused on morphological growth, and biomass allocation pattern^42–46^ or the non-beneficial effect of shading^47^. This non-beneficial effect of shading was illustrated in *Plukenetia volubilis* L.^48^, *Phlox drummondii* Hook.^49^ and *Opuntia humifusa* Raf.^50^ with a delay in flowering, and in *Olea europaea* L. that exhibited a reduction of individual fruit weight and oil concentration^51^.

In some species [e.g. *Nothofagus glauca* (Phil.) Krasser (hualo), *Picea sitchensis* (Bong.) Carr., *Thuja plicata*, Donn ex D. Donn], the response to shading varied from one trait to the other^44,52^. But our findings revealed a consistent beneficial effect of shade on most vegetative and growth traits. For instance, we found that height in shaded seedlings was at least twofold higher than those in sun-exposed seedlings. In addition, shaded seedlings presented larger leaf blade areas than sun-exposed seedlings. Overall, we found that *S. dulcificum* is an intermediate shade tolerant species. *Synsepalum dulcificum* successfully survived, grew and reproduced under moderate shading. According to the definition of Martínez-García *et al*.^47^, *S. dulcificum* can be deemed shade tolerant.

Our study also demonstrated a positive effect of compost application on the reproductive performance of the tree, implying that organic fertilization is as good as inorganic fertilization^34^ in accelerating bud, flower and fruit induction. This offers farmers the possibility to utilise locally available resources for the production of healthy fruits for a growing organic fruit market^53^. Compost application also consistently improved vegetative growth in *S. dulcificum*. This indicates that the species may be more responsive to fertilization than other fruit tree species such as *Uapaca kirkiana* (Muell. Arg.), and *Sclerocarya birrea* (Hochst.)^35^ where the effect of compost supply was not conclusive.

We observed that shoot biomass was higher in shaded seedlings than in seedlings that grew under full sunlight. Similarly, in non-fertilized seedlings, more biomass was allocated to roots, probably to better explore the soil for nutrients^34^. Biomass partitioning in plants is species-specific and dependent on the environment^54^. For instance, woody species increased their leaf mass fraction in response to waterlogging, while herbaceous species did not. According to the balanced growth hypothesis^55^, plants allocate their biomass in an optimal pattern if the required above- and below-ground resources limits growth to an equal extent. As such, resource allocation will then favour above-ground organs when light is limiting, whereas below-ground organs will be favoured when nutrient or water are the limiting factors.

Valladares and Niinemets^56^ indicated that in intermediate shade-tolerant species, stem elongation was often found to be the most plastic trait. Our observations also revealed seedling height as the most plastic trait in *S. dulcificum*. This supports the hypothesis that *S. dulcificum* is an intermediate shade-tolerant species. We observed that morphological traits in *S. dulcificum* were more plastic than fitness and biomass allocation traits irrespective of the factor into play. This plasticity pattern differed from what was reported on other species in the literature. For example, in *Quercus ilex* and *Phillyrea latifolia* L., the most plastic traits to light were the physiological traits^57^. Similarly, physiological traits were found more plastic than morphological traits in *Betula alleghaniensis* Britt^58^.

Our findings illustrate phenotypic plasticity of *S. dulcificum* to light exposure and to nutrient availability with potential implications in terms of evolution, adaptation and development in new environments^59^, especially in the context of climate change. The adaptive plasticity of *S. dulcificum* under reduced light exposure and nutrient-rich conditions highlights the potential of the species to successfully colonize new environments. This adaptive plasticity might partly explain the current distribution of the species. Indeed, the species naturally occurs in six African countries, of which four are humid forest countries (Congo, Cameroon, Nigeria, Ghana) and two are low forest cover countries (Benin and Togo)^60,61^. *Synsepalum dulcifucum* was recorded among the most common understorey species in the Littoral Congo forest in Congo (a humid forest country)^62^. In Benin and Togo (low forest cover countries), the species was rather characterized by a rarity index of 0.98^63^.

*Synsepalum dulcificum* is commercially important and has the potential to contribute to income generation, empowerment of smallholder farmers^29^, and overall economic growth of occurrence countries. For these reasons, the potential of the tree to withstand climate change is worthy of consideration. Climate change is undoubtedly a major threat to terrestrial ecosystems^64^, and it is postulated that in response to changing environmental conditions plants will either migrate to maintain their adaptive optimum, or respond through phenotypic plasticity^65^ and or adaptive evolution^66^. While phenotypic plasticity was often viewed as an immediate response to very rapid environmental changes, adaptive evolution was considered important over the long term^67^. *Synsepalum dulcificum* occurs naturally in West Africa and it is predicted that temperatures in that region will increase by up to 1 °C by 2066^67^, and conditions will be drier with the retraction of moist and wet zones^68^, as well as longer and more frequent heat waves^69^. Under these projections, the phenotypic plasticity to light exposure observed in the current experiment may become maladaptive due to the increased heat stress that will constrain the fitness of the species. It has been suggested that phenotypic plasticity holds the potential to help plant species to adapt to climate change only when the plasticity is adaptive under the newly experienced environment^70,71^. Previous findings in *S. dulcificum* indicated the crucial importance of water availability for growth and fitness in the species^34^. However, since it is predicted that the species will undergo higher water stress in its natural environment, we suggest that climate change will adversely affect the future distribution of *S. dulcificum* in at least West Africa, and more intensely in Benin where the species is currently distributed in the Guineo-Congolian region only (an area predicted to shift from wet to semi-arid conditions)^68^. The consequence is that adaptive evolution would be the most reliable mechanism for the species to withstand future climate change, while its success will hinge on the existence of an adequate level of genetic variation in the species. Moreover, adaptive evolution will need to be rapid enough to respond to climate change^66,72^. Therefore, the future development of *S. dulcificum* will require a change in the current cultivation system, from plain field production to a more appropriate future-climate resilient system. In this regard, our findings imply some agronomic and horticultural pathways including possible development of agroforestry systems, commercial propagation nursery establishment, and greenhouse orchard promotion.

Agroforestry systems are viewed as a climate-smart agriculture practice that enhances food security, while serving adaptation and mitigation objectives^73^, and are particularly suited to a context of increasing pressure on the land. For species such as *Theobroma cacao* L. and *Coffea arabica* L. that have been mostly cultivated as agroforests^74,75^, the use of intermediate shade to optimize the incomes of smallholder farmers and biodiversity services are now recommended^76,77^. This is relevant to *S. dulcificum*, which exhibits a high growth rate and reproductive performance under moderate shading and organic fertilization. As in *T. cacao*^75^, *S. dulcificum* is able to survive under either “service” legume shade trees (e.g. *Gliricidia sepium* (Jacq.) Walp) or “productive” shade tree crops (e.g. *Musa* spp, timber or other perennial tree crop species). *Synsepalum dulcificum* was known as a slow-growing species, in which growth can be improved with adequate water supply^28,34^. Our findings suggest that moderate shading and compost application are also favourable for the growth conditions of the species. All these characteristics combined together constitute a promising package to promote the greenhouse orchard development in the species, especially in a context of the emerging organic greenhouse production. Indeed some of the major constraints affecting profitability in fruit tree greenhouse promotion includes costs required to maintain light sources, ensure adequate light distribution, and intensive fertilization^78^. For a number of fruit tree species (e.g. *Pyrus communis* auct., *Acca selowiana* (O.Berg) Burret), shading created by either the competing individuals or the greenhouse structure often negatively affected growth^79^. For such species, external lighting source is vital for ensuring production and profit. In the case of *S. dulcificum*, there is minimal competition between individuals, and the species can tolerate moderate shading while requesting a limited amount of organic nutrient for optimal growth. Therefore, there is potential to cultivate the species in marginal areas of its distribution, provided a greenhouse technology exists.

Overall, we conclude that seedlings of *S. dulcificum* responded positively to shading and compost application. Shade and compost supply consistently improved vegetative growth, whereas the interaction between shade and compost supply accelerated transition to reproductive phase. Shaded and 4.5 g compost-fertilized seedlings started fruiting at 24-month-old. Biomass allocation in the species supported the balanced growth hypothesis and morphological traits exhibited higher plasticity. Shade induced higher plasticity than nutrient availability in the species. In both cases, plant height and stem diameter were among the most plastic traits. Based on the plasticity trends observed, we suggest *S. dulcificum* is an intermediate shade tolerant species and recommend the use of 4.5 g per seedling every two months, as the basic compost application dose.

## Methods

### Plant materials

In March 2015, mature and ripe fruits of *S. dulcificum* were hand–harvested from a single tree in the locality of Sèhouè, Benin (06°55′09.5″N, 002°16′23.3″E), and processed by removing their red outer skins and seeds. A total of 250 seeds were thus obtained and subsequently germinated in black polystyrene nursery bags (754.2 ml) filled with sterilized sowing substrate (see below) and using one seed per bag. The physico-chemical characteristics of the soil used as sowing substrate were as follows: pH_(KCl)_ = 5.48, pH_(H2O)_ = 5.88, silt = 25.75%, clay = 12.27%, sand = 61.98%, organic carbon = 1.03%, N = 0.06%, Mg = 2.37 meq/100 g, Ca = 0.63 meq/100 g, phosphorus = 2.08 meq/100 g, and assimilable phosphorus = 23.06 ppm. The germination process was conducted in the Laboratory of Genetics, Horticulture and Seed Science at the University of Abomey-Calavi, Benin (06°25′00.8″N, 002°20′24.5″E). Seeds germinated within 21–45 days after sowing, and the seedlings were grown in a nursery for 12 months before being used for this experiment. Seedlings were watered once a day to ensure an adequate supply of water to the growing medium.

### Experimental system

Vigorous, similarly-sized 12-month-old seedlings were selected and transplanted into black polystyrene pots (25 cm diameter; 15.26 L) filled with the same substrate used for germination, again with one seedling per pot. The experiment started in May 2016 after all seedlings were planted in their new containers.

Over the following 15-month period (May 2016–August 2017), we used a factorial split-plot to evaluate the effects of light exposure and compost application on the survival, vegetative growth, reproductive performance, biomass allocation, and plasticity of the transplanted *S. dulcificum* seedlings. Light exposure was the main plot factor, and seedlings were subjected to either full sunlight (Sun) or moderate shading (Shade). Compost application was the subplot factor with three levels: 0 g, 4.5 g, and 9.0 g of compost/seedling. To the best of our knowledge, there has been no study on the effect of organic nutrient supply (i.e. compost) on the performance of *S. dulcificum*. Furthermore, there has been no reliable study of the effect of organic fertilization in the Sapotaceae more broadly. Because there was no previous study that could be used as a reference for this study, we used compost dosages that would allow us to examine the plasticity of *S. dulcificum* in response to varying nutrient availability. Doses of 0 g, 4.5 g, and 9.0 g were chosen to represent a gradient of nutrient availability and were respectively defined as low, medium and high nutrient availability.

Each of the six treatment combinations was replicated three times, with each replicate consisting of 6–7 seedlings (i.e. 20 seedlings per treatment). Full sunlight represented the conditions of an open field, while the moderate shading simulated the light conditions in a tree-based system. The moderate shade was obtained using a shade-house built at 3.1 m height with an aluminium roof. The compost, applied per pot every two months from May 2016 to January 2017, was based on poultry organic manure with the following properties: pH_(H2O)_ = 6.9, nitrogen = 0.77%, phosphorus = 0.2%, potassium = 0.12, organic matter = 16.46%, organic carbon = 8.23%, magnesium = 0.42%, Calcium = 1.16%, and C/N ratio = 11. In total, five separate compost applications were made during the course of the study.

The climatic data during the experiment (May 2016–August 2017) are presented in Table S1. During the experiment, the monthly average temperature was 27.44 °C, relative humidity was 83.53%, total rainfall was 117.6 mm, and total solar radiation was 450 Mj/m^2^. These data were obtained from a weather station installed by the Trans-African Hydro-Meteorological Observatory (TAHMO) on the experimental site at the University of Abomey-Calavi and from the meteorological station of the International Institute of Tropical Agriculture (IITA), Abomey-Calavi, Benin, located less than 1 km from the experimental site.

### Measurements

We measured survival rate, growth (vegetative and reproductive), and biomass allocation in the treated seedlings. For survival rate, we counted the number of living seedlings in each replicate at the end of the experiment. For vegetative growth parameters, stem diameter, plant height, number of branches, and number of leaves were counted at the beginning and at the end of the experiment. Stem diameter was measured at the soil surface using a digital vernier caliper of 0.01 mm precision, and plant height was measured with a ruler from the soil surface to the tallest seedling point. For each seedling, we scanned three differently sized leaves (large, medium, and small) and transferred the images into the software Mesurim Pro Version 3.4 (Académie d’Amiens, Amiens, France) for leaf area (cm^2^) determination. The final leaf area for each seedling was the average value from the three harvested leaves^80^. The scanned leaves were afterwards oven-dried at 65 °C and then weighed to the nearest 0.01 g using a digital balance to obtain leaf dry mass. Specific leaf area (SLA) was afterwards calculated for each seedling as the leaf area divided by leaf dry mass.

For reproductive growth, we monitored the development of each seedling daily throughout the duration of the experiment. The number of plants bearing buds, flowers, and fruits, as well as the dates of first budding, flowering, and fruiting (where applicable) were noted. Proportions of budding, flowering, and fruiting seedlings were determined as well as the times to first budding, flowering, and fruiting.

At the end of the experiment, we selected three plants per treatment (one per replicate) for biomass allocation determination. Each selected plant was labelled and partitioned into leaves, stem, and roots; and each portion was oven-dried at 65 °C. We determined for each plant the root mass fraction (RMF = root dry mass/total plant dry mass), the stem mass fraction (SMF = stem dry mass/total plant dry mass), the leaf dry mass (LMF = leaf dry mass/total plant dry mass), and the root-to-shoot ratio [R/S = RMF/(LMF + SMF)].

### Statistical analysis

Growth rate, determined as the difference between measurements of growth parameters at the beginning and at the end of experiment, was used as a dependent variable. The effects of light exposure, compost application, and their interaction on seedling survival and on the proportion of seedlings bearing buds, flowers, and fruits were tested using generalized linear models fitted with a binomial/quasi-binomial error structure to account for over-dispersion. We used a two-factor ANOVA to analyse the effect of the interaction between light exposure and compost application on stem diameter, plant height, and biomass allocation (LMF, STM, RMF, and S/R). When compost application or the interaction between light exposure and compost application was significant, we used a contrast analysis to depict the effect of the increase in compost application. Based on significant factors, we determined the phenotypic plasticity index (PPI) per trait following the formula given by Valladares *et al*.^81^:

$${\rm{PPI}}=({{\rm{X}}}_{{\rm{\max }}}-{{\rm{X}}}_{{\rm{\min }}})/{{\rm{X}}}_{{\rm{\max }}}$$

with X_max_ and X_min_ corresponding respectively to the highest and the lowest values among the mean values of each factor’s modalities. PPI theoretically ranged from 0 (no plasticity of the trait to the considered factor/no response variation to the environmental gradient) to 1 (extreme plasticity and high sensitivity to the environmental factors). We used a one-way ANOVA or a t-test (where appropriate) to compare the plasticity among measured traits and functional groups (fitness, morphology, and biomass allocation) to the tested factors (light exposure and nutrient availability). All analyses were performed in R version 3.5.0^82^.

## Supplementary information

Supplementary file

## Data Availability

All data generated or analysed during this study are included in this published article (and its Supplementary Information files).
