# Supplementary material for: Shade and nutrient-mediated phenotypic plasticity in the miracle plant Synsepalum dulcificum (Schumach. & Thonn.) Daniell
Source: Sci Rep. 2019 Mar 26;9:5135. doi: 10.1038/s41598-019-41673-5 (PMC6435671; doi:10.1038/s41598-019-41673-5)
Supplement: Supplementary file 1 — Supplementary file [file 41598_2019_41673_MOESM1_ESM.pdf]

Shade and nutrient-mediated phenotypic plasticity in the miracle plant *Synsepalum dulcificum* (Schumach. & Thonn.) Daniell

Dèdéou A. Tchokponhoué[1*], Sognigbé N'Danikou[1], Jacob S. Houeto[1] and Enoch G. Achigan-Dako[1]

[1] Laboratory of Genetics, Horticulture and Seed Science (GBioS), Faculty of Agronomic Sciences (FSA), University of Abomey-Calavi (UAC), Benin

***Author for correspondence:** Dèdéou A. Tchokponhoué

Email: dedeoutchokponhoue@gmail.com; **Tel**: (+27) 769690165

Supplementary Information

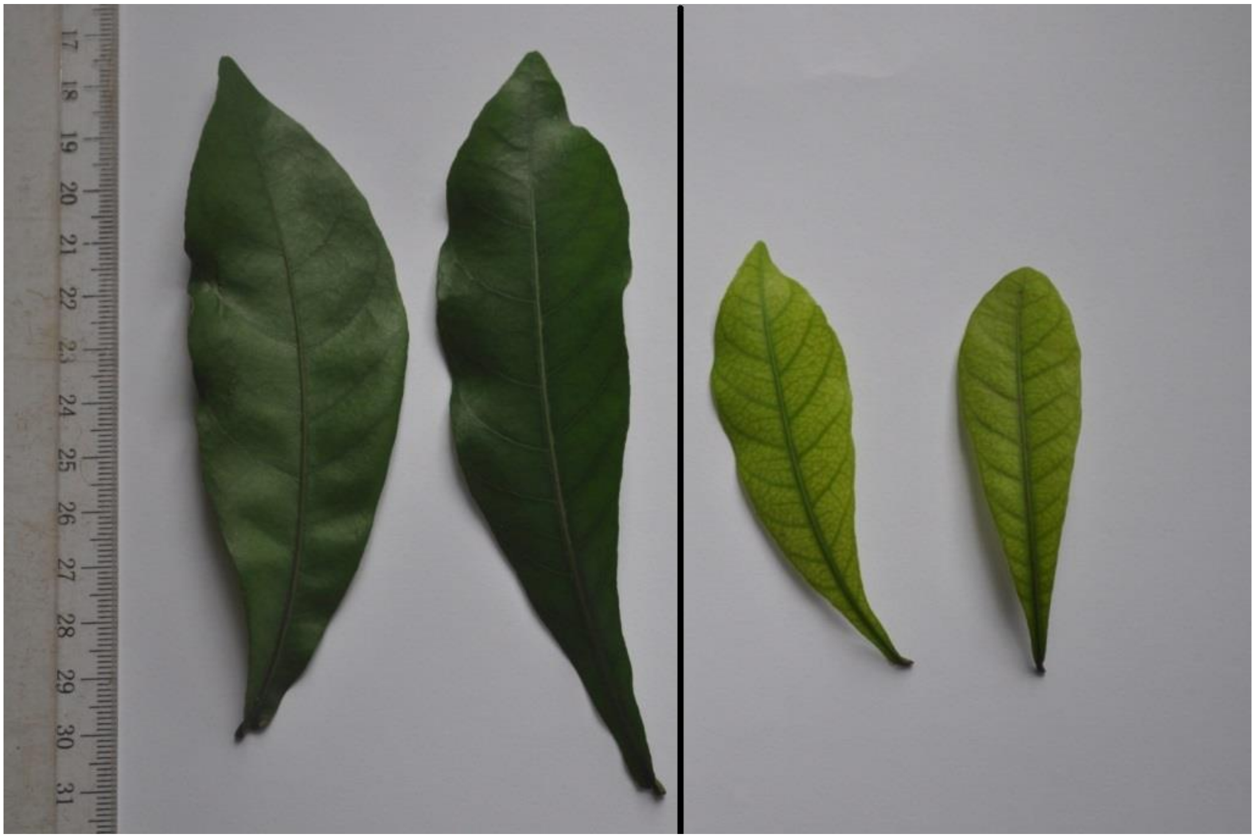

Fig. S1. Light induced plasticity in leaf colour in *Synsepalum dulcificum*. Shaded individuals (left) and full sun exposed individuals (Right).

Table S1. Total rainfall, solar radiation, monthly average temperature, and relative humidity during the experiment period (May 2016 – August 2017).

Year	Month	Total rainfall (mm)	Total radiation (Mj/m^2)	Average temperature (°C)	Average humidity (%)
2016	May	231.8	537.972	27.85	82.93
	June	127.8	382.11	26.41	86.87
	July	39.6	443.6	26.04	87.95
	Aug	23.6	423.27	25.8	86.69
	Sept	103.8	446.93	26.42	86.3
	Oct	326.6	466.19	28.7	85.02
	Nov	99	467.56	27.98	83.88
	Dec	0	455.47	28.2	78.8
2017	Jan	6.8	410.48	28.02	77.36
	Feb	5.2	393.31	29.02	78.14
	March	46.7	587.484	29.72	80.49
	April	100.8	548.45	28.94	82.03
	May	147.2	480.35	27.89	84.85
	June	378.6	404.43	26.69	87.89
	July	216.5	359.21	26.07	84.52
	Aug	29.1	402.2	25.43	82.82

Aug: August, **Sep**t: September, **Oct**: October, **Nov**: November, **Dec**: December, **Jan**: January, **Feb**: February.
